# Albiflorin alleviates cognitive dysfunction in STZ-induced rats

**DOI:** 10.18632/aging.203274

**Published:** 2021-07-28

**Authors:** Xiaojun Ma, Min Song, Yushan Yan, Gaofei Ren, Jingwen Hou, Guijun Qin, Wang Wang, Zhizhen Li

**Affiliations:** 1 Department of Endocrinology, The First Affiliated Hospital of Zhengzhou University, Zhengzhou 450052, Henan, China; 2 Department of Physiology and Neurobiology, School of Basic Medical Sciences, Zhengzhou University, Zhengzhou 450001, Henan, China

**Keywords:** albiflorin, cognitive dysfunction, Nrf-2 /HO-1/HMGB1/NF-κB pathway

## Abstract

Background: To explore the effect of albiflorin (AL) on streptozotocin (STZ)-induced Alzheimer's disease (AD) in rats.

Methods: A mouse model of diabetic encephalopathy was established by intraperitoneal injection of 1%STZ. Step down test and water maze test were used to test the cognitive function of rats. Congo Red Staining was used to detect the distribution of Aβ plaques in the hippocampus of rats. Cytokine levels in serum and hippocampus were measured using ELISA. Serum insulin, oral glucose tolerance (OGTT), serum superoxide dismutase (SOD) activity and malondialdehyde (MDA) content were measured by commercial kits. And the content of Nrf-2/HO-1/HMGB1/NF-kB in the hippocampus of diabetic rats were detected by western blot.

Results and Conclusion: Compared with the STZ model group, the average escape latency of rats in the AL group in the Morris water maze test was significantly shortened, and the average number of platform crossings and the ratio of distance/total swimming distance in the target quadrant were increased significantly. Staining of tissue sections and ELISA showed a decrease in Aβ plaque density in the hippocampus of rats in the AL group. And serum insulin levels of rats in the ALgroup were significantly reduced and OGTT was improved. In addition, AL could also regulate the Nrf-2/HO-1/HMGB1/NF-kB signal pathway in the hippocampus. Therefore, AL may ameliorate STZ-induced cognitive impairment in rats by regulating oxidative stress and inflammation in the brain.

## INTRODUCTION

Diabetes can cause a variety of complications, such as nephropathy, neuropathy and retinopathy. Recent studies have shown that diabetes also has some central nervous system complications [1]. This central nervous system complication of diabetes has been described in many clinical and experimental studies and is called diabetic encephalopathy. Diabetic encephalopathy can cause changes in biochemical properties, electrophysiology and morphology of hippocampal neurons in the brain [2]. These changes can cause cognitive defects in diabetic patients and seriously affect the quality of life [3]. At present, many studies at home and abroad focus on diabetic peripheral neuropathy, while the pathophysiological mechanism of neuropathy caused by diabetic encephalopathy has received less attention. However, the prevalence of cognitive dysfunction in diabetes will increase dramatically with longer survival time of diabetic patients. Therefore, it is urgent to clarify the pathogenesis of diabetic cognitive dysfunction and find effective prevention and treatment strategies.

AL, a monoterpenoid glycoside, is one of the active ingredients of *Paeonia lactiflora*, a commonly used traditional Chinese medicine. In recent years, with the in-depth study of pharmacodynamics of paeoniflorin, it has been discovered that AL has unique pharmacodynamic characteristics different from paeoniflorin, and is superior in blood, antidepressant and potential treatment of diabetes [4–6]. It also has pharmacodynamic effects such as analgesia, anticonvulsant, anti-inflammatory and hepatoprotective [7]. In addition, clinical studies have shown that it has a good anti-inflammatory effect on some inflammatory diseases such as enteritis and rheumatoid arthritis [8, 9]. Therefore, on the basis of STZ-induced dementia in rats, this study investigated the effects of AL on diabetic rats.

## RESULTS

### AL decreased serum insulin level and improved OGTT

**Figure 1:**
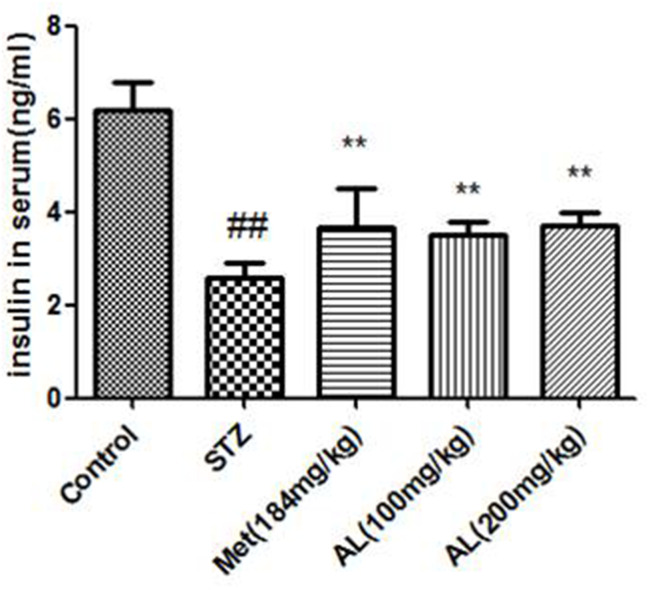
**AL decreased serum insulin level in rats.** Values are expressed as means±SDs. Compared with control: ^#^ P<0.05, ^##^P<0.01; Compared with model:*P<0.05, **P<0.01.

**Figure 2:**
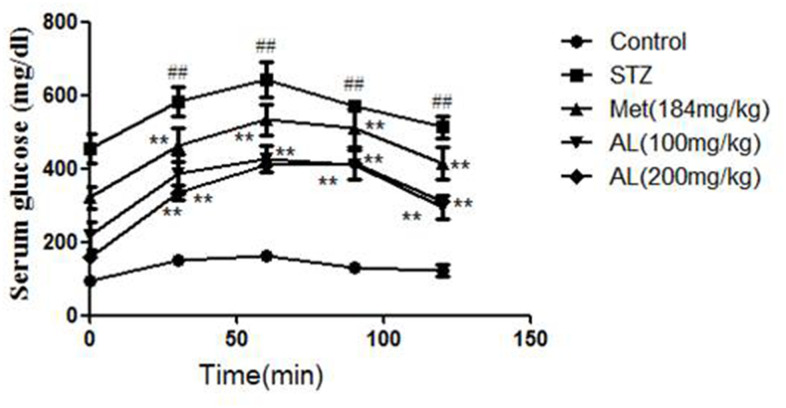
**AL improved OGTT in rats.** Values are expressed as means±SDs. Compared with control: ^#^ P<0.05, ^##^P<0.01; Compared with model:*P<0.05, **P<0.01.

Compared with the control group, the serum insulin in the model group was significantly decreased. As expected, AL and Met markedly increased the serum insulin level. In OGTT experiment, the blood glucose of model group rats was higher than that of control group rats within 30, 60 and 90 minutes, and the above changes were obviously changed by AL and Met (Figures 1, 2).

### Step down experiment and Morris water maze test

**Figure 3:**
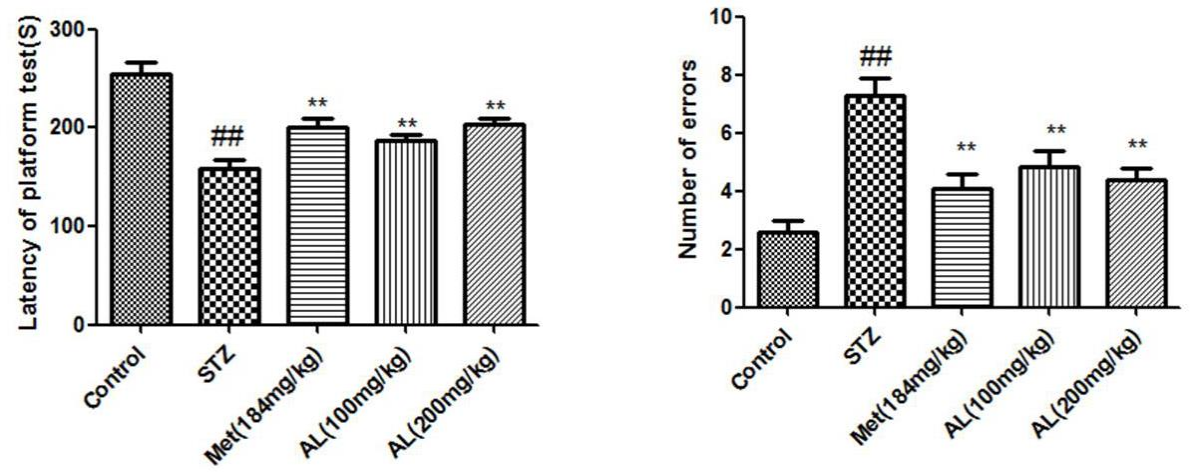
**Step down experiment in rats.** Values are expressed as means±SDs. Compared with control: ^#^ P<0.05, ^##^P<0.01; Compared with model:*P<0.05, **P<0.01.

**Figure 4:**
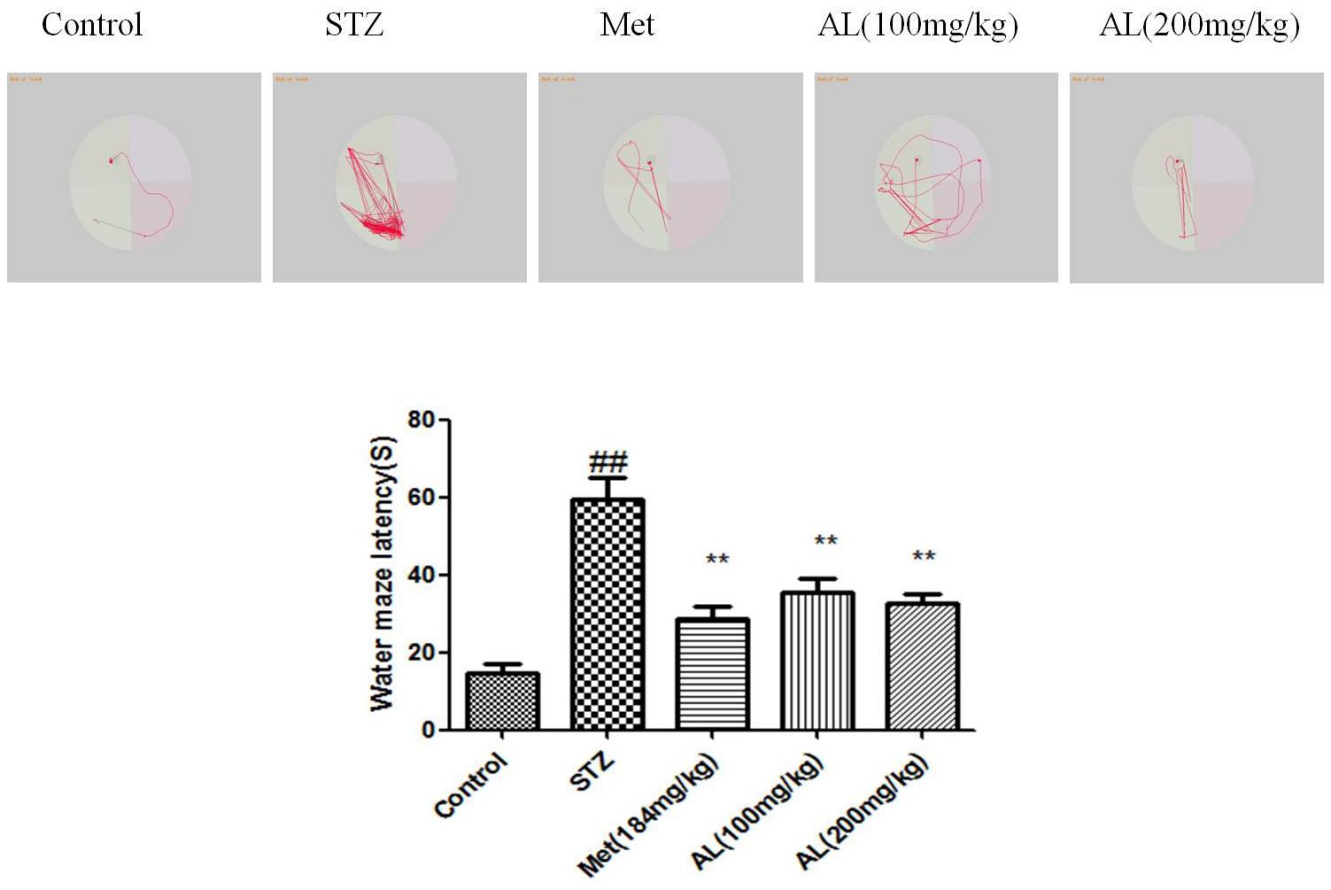
**Morris water maze test in rats.** Values are expressed as means±SDs. Compared with control: ^#^ P<0.05, ^##^P<0.01; Compared with model:*P<0.05, **P<0.01.

In the platform test, compared with the control group rats, the latent period (seconds) of the model group was significantly shortened, while the latent period of the AL and Met groups rats was significantly increased. Relative to the control group, the numbers of errors in the model group were significantly increased, while the number of errors in the AL and Met groups decreased markedly. Compared with normal group rats, the latency of model group rats was significantly reduced, AL and Met can obviously increase the latency of rats (Figures 3, 4).

### SOD activity and MDA content in serum

**Figure 5:**
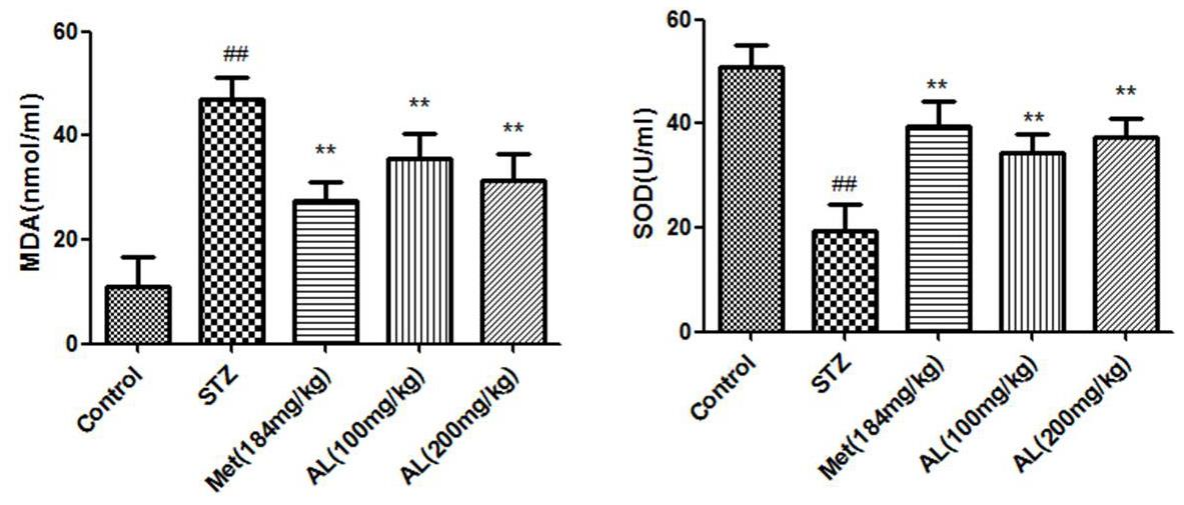
**SOD activity and MDA content in serum.** Values are expressed as means±SDs. Compared with control: ^#^ P<0.05, ^##^P<0.01; Compared with model:*P<0.05, **P<0.01.

Compared with the normal rats, the serum SOD activity and MDA content in the model group were significantly decreased. However, AL and Met significantly increased the serum SOD activity and decreased the MDA content in the rats (Figure 5).

### Cytokines in serum and hippocampus

**Figure 6:**
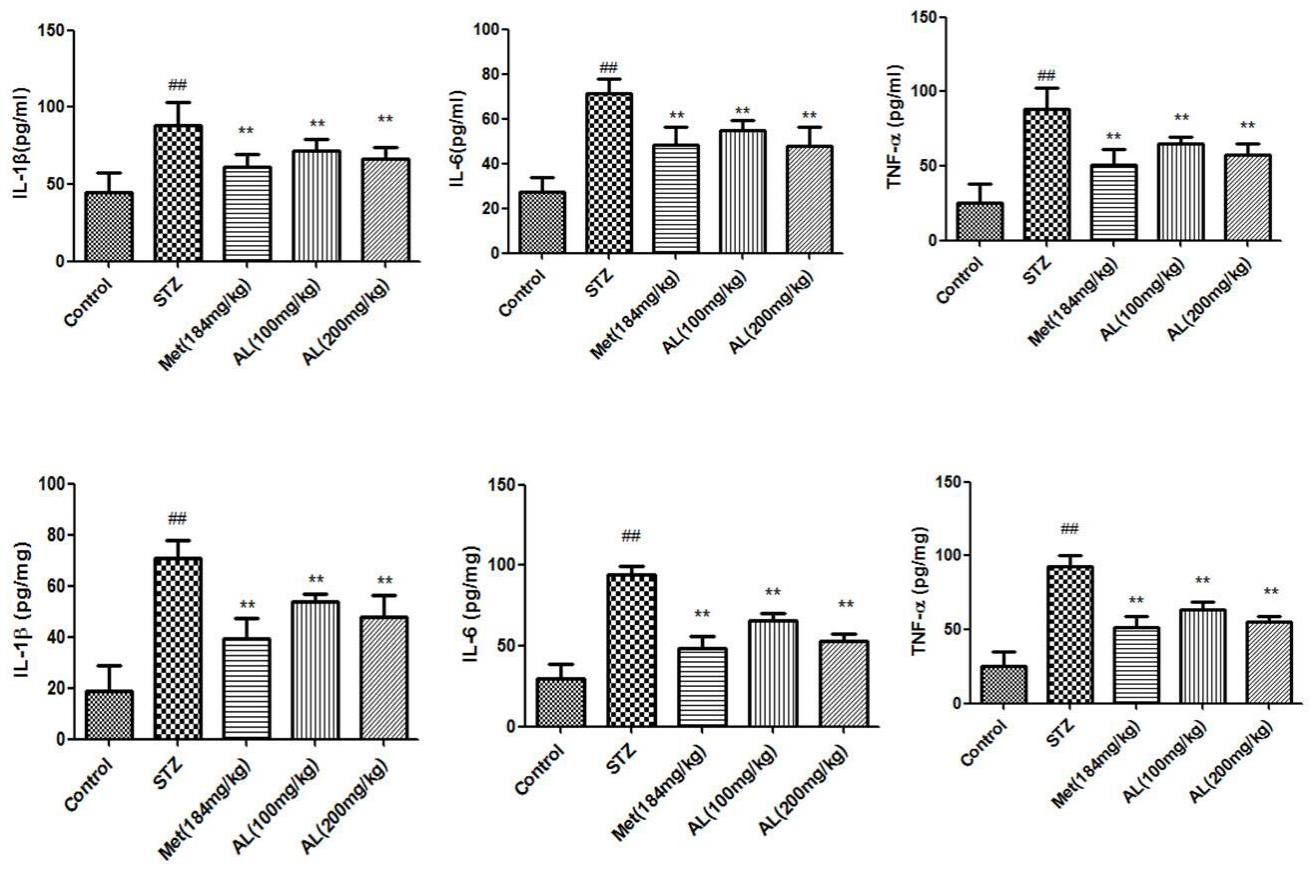
**Cytokines in serum and hippocampus.** Values are expressed as means±SDs. Compared with control: ^#^ P<0.05, ^##^P<0.01; Compared with model:*P<0.05, **P<0.01.

Compared with normal rats, cytokines (TNF-α, IL-1β and IL-6) in serum and hippocampus of model rats increased significantly. As expected, ALT and Met significantly reduced cytokines (TNF-α, IL-1β and IL-6) in serum and hippocampus of rats (Figure 6).

### Effects of AL on Nrf-2 /HO-1/HMGB1/NF-κB pathway in hippocampus

**Figure 7:**
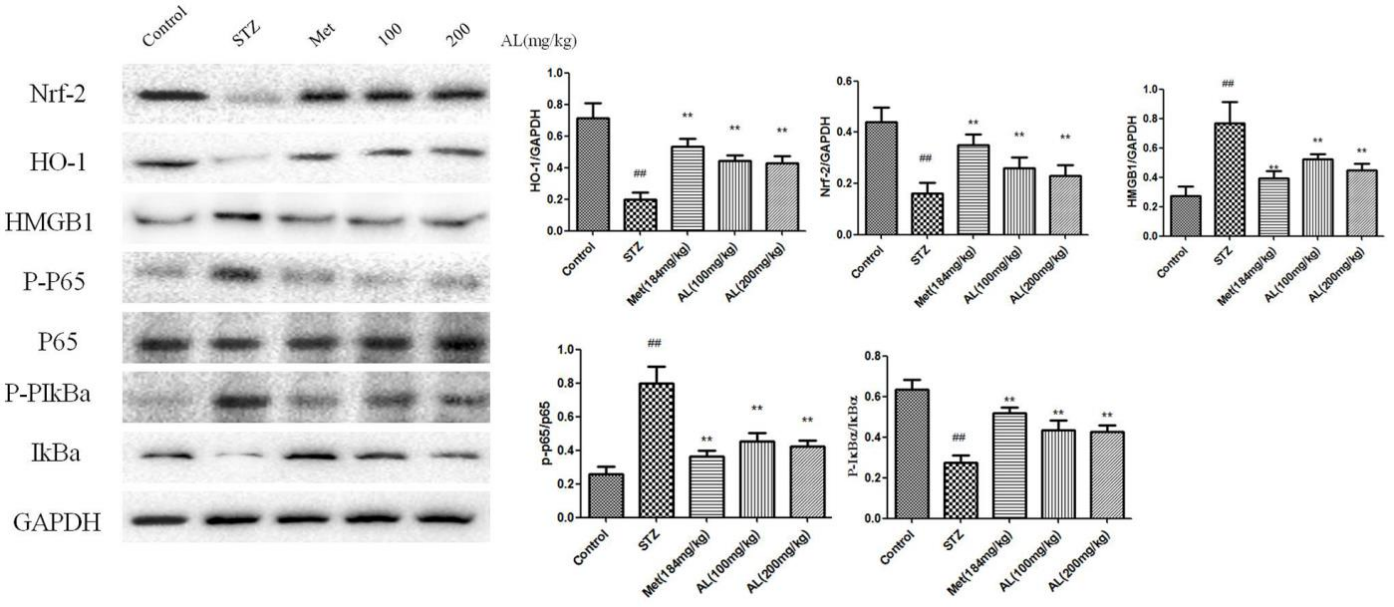
**Effects of AL on Nrf-2 /HO-1/HMGB1/NF-κB pathway in hippocampus.** Values are expressed as means±SDs. Compared with control: ^#^ P<0.05, ^##^P<0.01; Compared with model:*P<0.05, **P<0.01.

Compared with control rats, the levels of Nrf-2 and HO-1 in the hippocampus of rats in the model group were significantly reduced, while the levels of HMGB1, p-NF-kBP65 and p-IkBa were significantly increased. As expected, ALT and Met significantly increased the levels of Nrf-2 and HO-1 in the hippocampus of rats, and reduced the levels of HMGB1, p-NF-kBP65 and p-IkBa in the hippocampus of rats (Figure 7).

### Effects of AL on Nrf-2 and HMGB1by immunohistochemistry analysis

**Figure 8:**
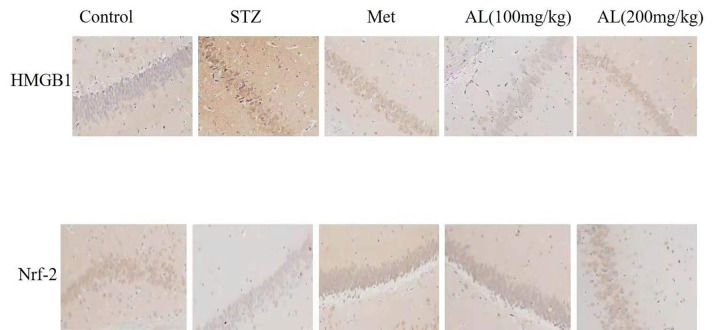
Effects of AL on Nrf-2 and HMGB1 by immunohistochemistry analysis(x100).

Compared with control rats, the levels of Nrf-2 in the hippocampus of rats in the model group were significantly reduced, and the levels of HMGB1 were significantly increased. While ALT and Met significantly increased the levels of Nrf-2 and reduced the levels of HMGB1 in the hippocampus of rats when compared with the control group (Figure 8).

### Observation of hippocampus by transmission electron microscope

**Figure 9:**
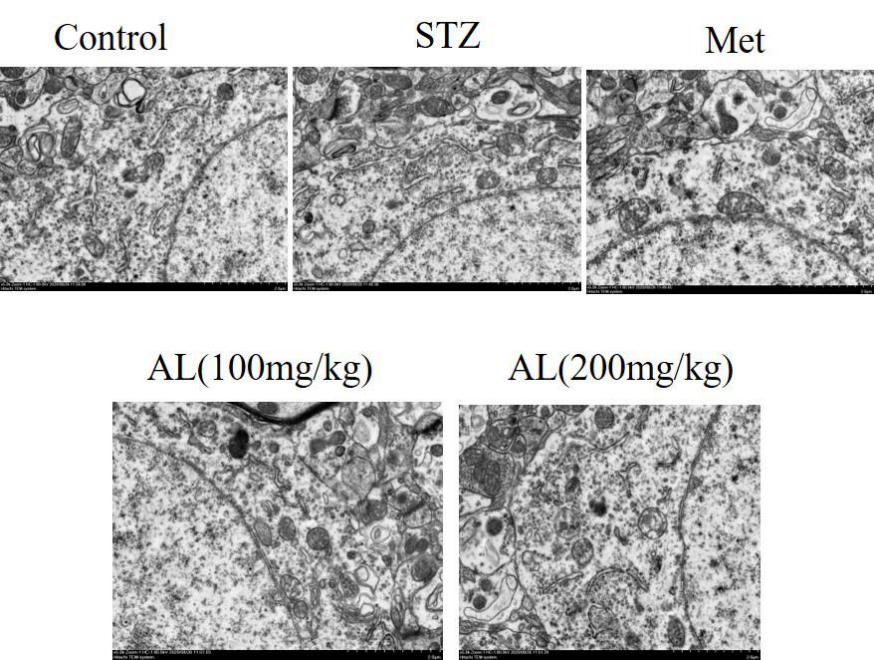
Observation of hippocampus by transmission electron microscope (x400).

The differences in nuclei and organelles among the 3 groups were observed by transmission electron microscopy. The results showed that no morphological changes and apoptosis occurred in the CA1 neurons of the hippocampus in the control group, while a large number of cells in the model group underwent apoptosis due to morphological changes. Compared with the model group, after the treatment of AL, the diabetic rats had larger nuclei, smoother nuclear membrane, and normal cell morphology (Figure 9).

### Number of neurons in hippocampal CA1

**Figure 10:**
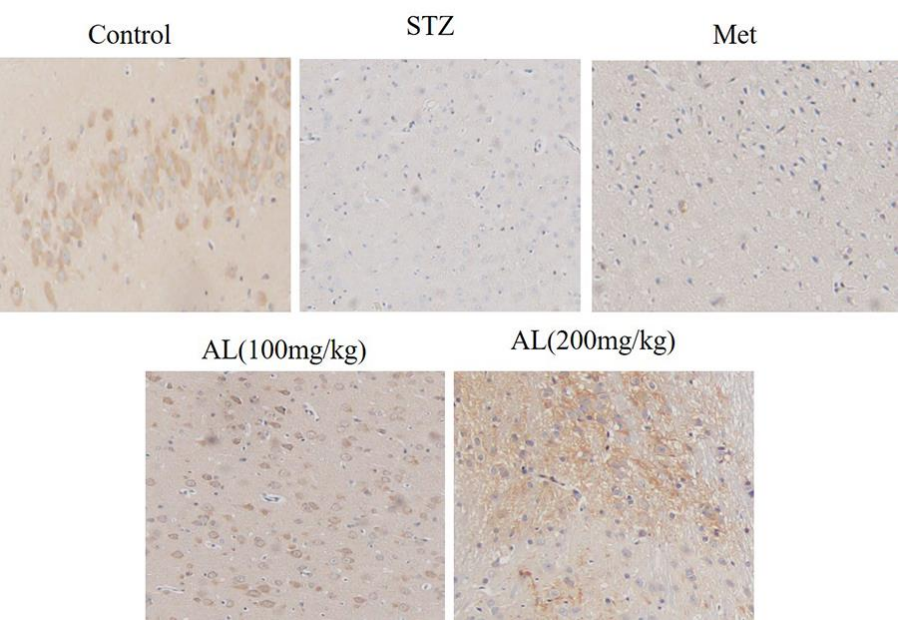
Number of neurons in hippocampal CA1(x100).

Compared with the normal group, the neuron shrinkage, loss, and loose arrangement were observed in the hippocampal CA1 region in the model group. While in the AL group, the damage caused by diabetes had recovered (Figure 10).

## DISCUSSION

Diabetes is a chronic metabolic disease caused by hyperglycemia, which may cause various physiological complications. There is currently no cure for diabetes, which is a worldwide problem. At present, the therapeutic drugs for diabetes, such as insulin and other hypoglycemic agents can only relieve the symptoms of hyperglycemia, and there are certain side effects in the process of treatment. Chinese herbal medicine or other adjuvant treatments have similar effects compared with western medicine, which is welcome and the toxic side effect of Chinese medicine is very small or non-toxic [10, 11]. Our study found that AL could significantly ameliorated OGTT, increase serum insulin levels, decrease the content of cytokines in serum and hippocampus, reduce serum MDA, increase serum SOD activity, and improve cognitive behavior in diabetic rats, which may be achieved by regulating Nrf-2/HO-1/HMGB1/NF-κB signaling pathway in hippocampus.

Many literatures indicate that inflammation is involved in the pathological process of cognitive dysfunction [12]. Hyperglycemia stimulates the production of TNF-α, which increases vascular permeability and neuronal damage, and aggravated cognitive impairment [13]. Some studies suggest that that cytokines are involved in cognitive dysfunction caused by multiple factors [14]. In this study, we found that AL could significantly reduce the levels of cytokines in the serum and hippocampus of diabetic rats.

Free radical-induced oxidative stress is a common phenomenon, which plays an important role in brain cognitive impairment and is a key factor. Oxidative stress increases neuronal membrane lipid peroxidation, which leads to neuronal damage and apoptosis [15]. Oxidative stress is involved in the pathological process of cognitive impairment caused by diabetes, which makes anti-oxidation an important means of neuroprotection [16, 17]. Excessive oxidative stress in diabetic organisms that damages nerve cells. MDA is a well-established marker of oxidative stress and can reflect the degree of oxidative stress in the body [18]. SOD is an important enzyme that clears free radicals in the body. This experiment showed that AL significantly reduced the serum MDA content and increased the SOD activity in diabetic rats.

Nrf2 is an important antioxidant protein, which can resist inflammation and oxidative stress caused by many factors, and promote the expression of multiple antioxidant genes. And the activation of Nrf-2 is regulated by Keap-1 [19]. Under the stimulation of external oxidative stress, Nrf-2 is released from Keap-1 and binds to antioxidant-related elements (ARE), which increases the expression of NAD (para) hydroquinone oxidoreductase (NQO1) and HO-1, which in turn improves the effect of anti-inflammatory and antioxidant in the body [20]. HO-1 is an important protein involved in inflammation and oxidative stress. Nrf2/HO-1 activates NF-κB and regulate cytokine expression [21]. High-mobility group box 1 (HMGB1) expressed as single 215 amino acids polypeptide chain and consists of three structural domains. It was firstly identified as a high conserved transcription factor presented in the nucleus to maintain the nucleosome structure and regulate gene transcription, and later confirmed to be a cytokine which is released by injured cells or monocytes and macrophages. Previous studies showed that HMGB1 also participate in lipid metabolism, and atorvastatin could reduce IL-6 and TNF-α levels by inhibiting plasma HMGB1 [22–24]. In this study, ALT significantly increased the levels of Nrf-2 and HO-1 in the hippocampus of rats, and reduced the levels of HMGB1, p-NF-κBP65 and p-IkBa in the hippocampus of rats.

## CONCLUSIONS

In summary, AL can improve diabetes-induced cognitive dysfunction, and its mechanism is related to the regulation of hippocampal Nrf-2/HO-1/ HMGB1/NF-κB signaling pathway, and its in-depth mechanism needs further study.

## MATERIALS AND METHODS

### Reagents and instruments

AL and STZ were obtained from Sigma. BCA Protein Quantitative Kit (Bosch, Wuhan). Congo red dye kit (Solarbio, Beijing). Morris Water Maze Palace was purchased from Anhui Zhenghua Biological Equipment Co., Ltd. and Ethovision 3. 0 video tracking and processing system was purchased from Dutch company Wageningen. Glucose was obtained from Seagate, France. SOD and MDA kits were produced by Jiancheng Institute of Biotechnology (Nanjing, China). Blood glucose test paper was purchased from Shanghai Jinque Biology Company. Cytokine kits was purchased from Beijing Tianlai Biotechnology Co., Ltd. All antibodies were purchased from Santa Cruz Biotechnology Co., Ltd.

### Animals

100 SD rats, male, 2 months old, weighing 200-250 g, were provided by experimental animal center of Zhengzhou university. Therefore, animal experiments were conducted in accordance with the animal ethics guidelines of Zhengzhou University.

### Experimental protocol

This study included 100 male Wistar rats. Among them, 80 rats were taken as diabetic models and fed with high-fat diet one week after adaptive feeding. At the same time, 20 rats were taken as control group and fed with normal diet. Four weeks later, rats in diabetes model group were injected with STN in the tail vein injection of STZ, while rats in control group were given citrate buffer. Whole blood glucose and plasma insulin levels were monitored one week after STZ or citrate buffer injection. Rats were excluded from the next study if the high fat diet combined with STZ injection was unsuccessful (fasting plasma glucose ≥ 11.2 mmol/L after one week of STZ injection was considered successful). Finally, the final number of STZ injected rats meeting the requirements of the model was 72. Meanwhile, diabetic rats meeting the requirements of the model were divided into four groups: model group, model + metformin (184 mg/kg) group, model + AL (100 mg/kg) group and model +AL (200 mg/kg) group. The corresponding drugs were administrated by gastric gavage for 10 weeks. Rats in the control group were given normal diet and drinking water at will, while rats in the STZ treatment group were fed high-fat diet until the end of the 16th week.

### Oral glucose tolerance test (OGTT)

Rats were fasted for 16 hours and then fed with 30% glucose (1.5 g/kg). Blood was collected from caudal vein at 0, 30, 60, 90, and 120 min. Samples were centrifuged at 3000 × g for 8 minutes at 4° C, and the serum obtained was detected by automatic biochemical detector.

### Detection of serum insulin

Using One Touch Ultra Link blood glucose meter (Life Scan, China), 3-μl blood drops obtained through tail amputation were used to measure blood glucose level. According to the instructions (Millipore, EZRMI-13, U.S.A).

### Hippocampus and serum cytokines levels

The levels of serum and hippocampal cytokines were determined by sandwich enzyme linked immunosorbent assay (ELISA), and the determination method was referred to the kit instructions.

### SOD and MDA in serum

Superoxide dismutase (SOD) activity in serum was determined by inhibition of autoxidation of pyrogallol. The concentration of MDA was calculated by the reaction between thiobarbituric acid in serum and malondialdehyde as substrate.

### Evaluation of cognitive function by step-down test

The size of the platform test box was 25 cm × 25 cm × 75 cm, the bottom of the box was covered with a metal grid, and a 5 cm high and 8 cm diameter safety platform was placed in the corner of the bottom of the box. On the first day, the rat was placed on the safety platform. When the rat jumped off the platform, its limbs would be hit by electricity (3 Hz; 0. 4 m A), and after repeated jumps up and down, the rat would eventually stop on the platform for 300 s. The same experiment was repeated on the second day, and the time (latency period) when the rat first jumped out of the platform and the number of times that the rat jumped out of the platform within 300 s (number of errors) were recorded.

### Morris water maze experiment to evaluate cognitive function

Morris water maze test was performed after the platform jumping test to evaluate the learning and memory ability of each group of rats. Morris Water Maze was a round stainless steel pool with a diameter of 150 cm and a height of 60 cm. The pool was divided into 4 quadrants. The escape platform was a transparent, circular cylindrical platform with a diameter of 10 cm. Its surface was 2 cm from the water surface. Place the platform in any quadrant during the test. Selected a quadrant and placed the rat facing the wall in the water. When the rat arrived at the platform, it was allowed to stay on the platform for 30 s before continuing with the next training. The time to find the platform did not exceed 60 s. And the rats were trained 4 times a day, and we recorded the time (latency) that the rats took to reach the platform each time, for a total of 5 days.

### Western bolt

The total protein of frozen brain tissue was extracted and subjected to SDS-polyacrylamide gel electrophoresis; the sample protein was transferred to a polyvinylidene fluoride (PDVF) membrane; the blocking solution was blocked for 1 h; the primary antibody diluted by the blocking solution was added and shaken overnight at 4° C; washed with TPBS 3 times, added fluorescently labeled secondary antibody, and shaken in the dark for 1 h in the greenhouse; image analysis was performed after scanning with TPBS.

### Immunohistochemistry

Slices were dehydrated and dewaxed, and washed with PBS for 3 times, 5 min each time, microwave repairing in EDTA buffer solution, power off after medium fire to boiling, low fire to boiling at intervals of 10 min, washed with PBS for 3 times, 5 min each time after cooling, incubated in 3% hydrogen peroxide solution at room temperature in dark for 10 min, washed with PBS for 3 times, 5 min each time, blocked with 5% BSA for 20 min after spin-drying, then removed BSA solution, adding diluted primary antibodies to cover tissues, overnight at 4° C, washed with PBS for 3 times, 5min each time, removed PBS solution, and added II antibody at 37° C, incubated for 30 min, washed with PBS for 3 times, 5 min each time, DAB color development, hematoxylin redyed, dehydration, and sealed with neutral gum. The images were analyzed by Image-Pro Plus 6. 0 image analysis software and the mean absorbance (ma) value of each sample was calculated.

### Ultrastructure of hippocampal CA1 cells observed by transmission electron microscope

Several pieces of 1 mm^3^ tissue were taken from hippocampal CA1 region of each group of experimental animals, fixed with 2.5% glutaraldehyde for 24 h, and then fixed with 10% osmium tetroxide for more than 24 h. ethanol was dehydrated step by step, then embedded with epoxy resin, and sliced with ultrathin slicer. uranium acetate/lead citrate were double dyed, observed under transmission electron microscope, and photographed with 5000 times magnification (JEM-2000EX transmission electron microscope, Japan).

### Density count of neurons in hippocampal CA1 region

The paraformaldehyde-fixed hippocampus of each rat was subjected to continuous coronal section (4-6 m) and H&E staining. According to Paxinos and Watson's study, the hippocampus was divided into several regions, such as CA1-CA4, SC (subiculum) and dentate gyrus. Olympus microscope was used to take photos, and an ultra-micro analysis system was used to analyze and count. The number of surviving neuron cells per square millimeter was defined as neuron cell density.

### Statistical analysis

SPSS19.0 statistical software was used to analyze the data, and the measurement data were expressed as mean ± SD; T test was used for comparison between the two groups, and nonparametric test was used for nerve injury score analysis. The difference was statistically significant with p < 0.05.

### Availability of data and materials

All the dataset and materials analyzed during this study were available.

### Animal ethics approval

All animal experiments were conducted in accordance with animal ethics of Zhengzhou University.
